# A Brain Computer Interface Neuromodulatory Device for Stroke Rehabilitation: Iterative User-Centered Design Approach

**DOI:** 10.2196/49702

**Published:** 2023-12-11

**Authors:** Gemma Alder, Denise Taylor, Usman Rashid, Sharon Olsen, Thonia Brooks, Gareth Terry, Imran Khan Niazi, Nada Signal

**Affiliations:** 1 Rehabilitation Innovation Centre, Health and Rehabilitation Research Institute Auckland University of Technology Auckland New Zealand; 2 Centre for Chiropractic Research, New Zealand College of Chiropractic Auckland New Zealand; 3 Sensory Motor Integration, Department of Health Science and Technology, Aalborg University Aalborg Denmark

**Keywords:** user-centered design, stroke, rehabilitation technology, wearable technology, brain computer interface, BCI, mobile app, think-aloud, near live, semistructured interviews

## Abstract

**Background:**

Rehabilitation technologies for people with stroke are rapidly evolving. These technologies have the potential to support higher volumes of rehabilitation to improve outcomes for people with stroke. Despite growing evidence of their efficacy, there is a lack of uptake and sustained use in stroke rehabilitation and a call for user-centered design approaches during technology design and development. This study focuses on a novel rehabilitation technology called exciteBCI, a complex neuromodulatory wearable technology in the prototype stage that augments locomotor rehabilitation for people with stroke. The exciteBCI consists of a brain computer interface, a muscle electrical stimulator, and a mobile app.

**Objective:**

This study presents the evaluation phase of an iterative user-centered design approach supported by a qualitative descriptive methodology that sought to (1) explore users’ perspectives and experiences of exciteBCI and how well it fits with rehabilitation, and (2) facilitate modifications to exciteBCI design features.

**Methods:**

The iterative usability evaluation of exciteBCI was conducted in 2 phases. Phase 1 consisted of 3 sprint cycles consisting of single usability sessions with people with stroke (n=4) and physiotherapists (n=4). During their interactions with exciteBCI, participants used a “think-aloud” approach, followed by a semistructured interview. At the end of each sprint cycle, device requirements were gathered and the device was modified in preparation for the next cycle. Phase 2 focused on a “near-live” approach in which 2 people with stroke and 1 physiotherapist participated in a 3-week program of rehabilitation augmented by exciteBCI (n=3). Participants completed a semistructured interview at the end of the program. Data were analyzed from both phases using conventional content analysis.

**Results:**

Overall, participants perceived and experienced exciteBCI positively, while providing guidance for iterative changes. Five interrelated themes were identified from the data: (1) “This is rehab” illustrated that participants viewed exciteBCI as having a good fit with rehabilitation practice; (2) “Getting the most out of rehab” highlighted that exciteBCI was perceived as a means to enhance rehabilitation through increased engagement and challenge; (3) “It is a tool not a therapist,” revealed views that the technology could either enhance or disrupt the therapeutic relationship; and (4) “Weighing up the benefits versus the burden” and (5) “Don’t make me look different” emphasized important design considerations related to device set-up, use, and social acceptability.

**Conclusions:**

This study offers several important findings that can inform the design and implementation of rehabilitation technologies. These include (1) the design of rehabilitation technology should support the therapeutic relationship between the patient and therapist, (2) social acceptability is a design priority in rehabilitation technology but its importance varies depending on the use context, and (3) there is value in using design research methods that support understanding usability in the context of sustained use.

## Introduction

### Background

Stroke is a major health, socioeconomic, and financial burden that affects over 12 million people worldwide annually [1]. Despite advances in stroke prevention, the incidence of stroke is anticipated to rise due to population growth and aging [2]. Following a stroke, up to 80% of individuals experience difficulty with locomotion [3,4]. Locomotion refers to the ability to move from one place to another [5] and encompasses a wide range of activities such as getting on and off a chair; walking indoors; climbing stairs; and navigating obstacles, terrains, and environments. While most people with stroke regain some ability to walk unassisted, less than 20% achieve unrestricted community locomotion [6,7]. Rehabilitation can reduce locomotor disability following stroke, particularly when delivered in large volumes [8-14], yet observational studies confirm the amount of rehabilitation received is limited, translating into poorer outcomes for people with stroke and consequent lifelong disability [15-20]. Thus, innovative approaches for stroke rehabilitation are required.

The last 2 decades have witnessed the rapid development of rehabilitation technologies, such as robotics, virtual reality, neuromodulation devices, activity monitors, and mobile apps designed to augment rehabilitation after stroke. While there is evidence that these technologies can increase the amount of rehabilitation a person with stroke receives and improve outcomes [13,14,21-26], user adoption and sustained use of such technologies remains low [27-33]. The disconnect between initial efficacy and clinical translation likely relates to the usability of these technologies and their acceptability to users [34,35]. As a result, there has been a call for increased application of user-centered design approaches in the development of rehabilitation technologies [35-37]. Adopting user-centered design approaches can support the development of usable and acceptable technologies by prioritizing user needs, involving users and relevant stakeholders throughout the project life cycle, and modifying the design of the technology based on iterative user-centered evaluation [38,39].

Noninvasive neuromodulatory interventions are rehabilitation technologies with the potential to maximize rehabilitation outcomes and reduce physical disability. Typically, these interventions involve repeated magnetic or electrical stimulation of the central and peripheral nervous systems to induce neural plasticity [40]. Noninvasive neuromodulatory interventions that target movement control have the potential to accelerate stroke recovery when combined with traditional rehabilitation [41-44]. However, such interventions often rely on complex medical devices and user interfaces operated by expert operators, and lack usability and acceptability. To maximize the potential for successful implementation in rehabilitation practice, research and development of noninvasive neuromodulatory technologies must include a user-centered approach [45]. In this paper, we present a complex neuromodulatory rehabilitation technology (exciteBCI) and its evaluation in a user-centered design research process.

### exciteBCI

exciteBCI is a prototype, portable, medical wearable device designed to deliver neuromodulation during locomotor rehabilitation for people with stroke. The device uses a brain computer interface in which a specific electroencephalography signal, which reflects the person’s intention to move, is extracted and paired with the afferent stimulus from peripheral electrical stimulation [46,47]. The electrical stimulation is timed to coincide precisely with the electroencephalography signal in the motor cortex to induce neural plasticity [46,47].

exciteBCI evolved from an endeavor to translate a neuromodulatory intervention that had been tested in healthy and stroke populations in a clinical research laboratory setting [46-54] into a rehabilitation device suitable for stroke rehabilitation. Prior feasibility work found that the neuromodulatory intervention, when delivered during simple ankle movements while seated, was not acceptable to people with stroke and was not feasible for rehabilitation [55]. The equipment was deemed cumbersome and uncomfortable, the set-up time was excessive, and the movement tasks were considered meaningless and boring by people with stroke. Given that qualitative evidence indicates that rehabilitation should be centered on meaningful real-world activities that reflect a person’s aspirations and should be practiced at progressively higher intensities [56,57], these perspectives have important ramifications for the implementation of the intervention in clinical practice and for ensuring sustained use.

The iterative user-centered design process for developing exciteBCI was guided by the International Organization for Standardization (ISO) 9241-210:2010 standard [38] and was driven by a transdisciplinary team comprising physiotherapists, biomedical engineers, product designers, user experience and user interface designers, and a lived experience researcher. Initial work involved the following three phases: (1) understanding and specifying the context of use, (2) identifying user requirements, and (3) iteratively developing design solutions [58]. This paper reports the fourth stage of the ISO 9241-210:2010 standard [38]: (4) evaluating the design. The aims of this research were to (1) explore users’ perspectives and experiences of exciteBCI and how well it fits with rehabilitation, and (2) facilitate modifications to exciteBCI design features.

## Methods

### Study Design

**Figure 1:**
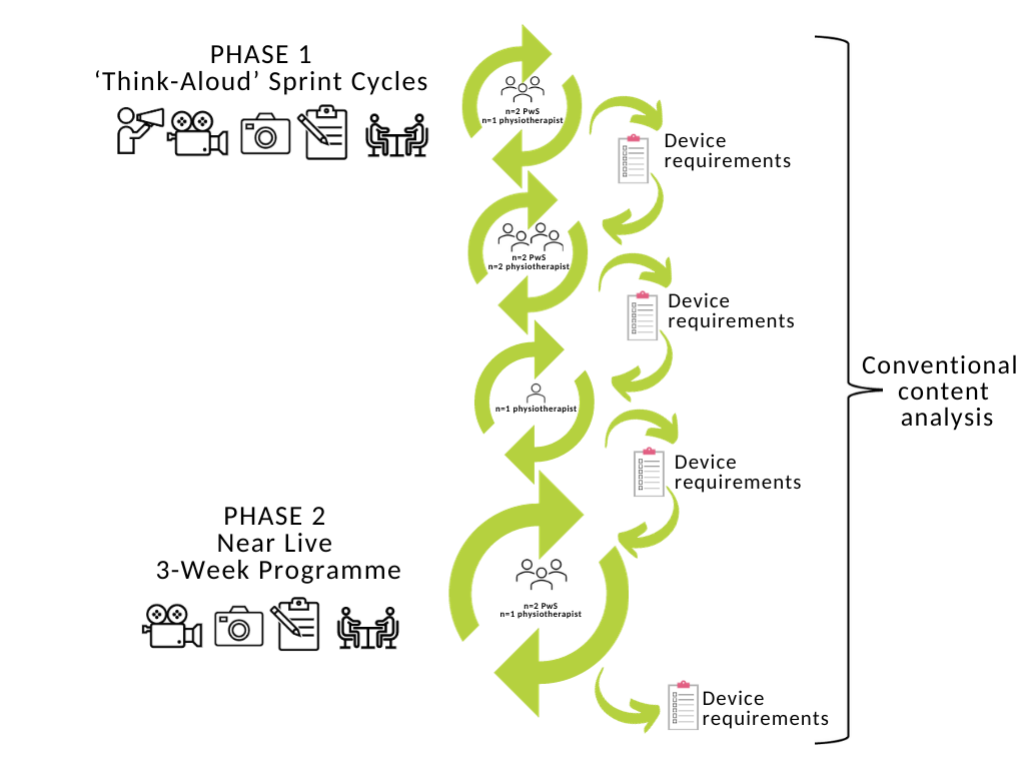
Overview of study design, data collection procedures, and data analysis. PwS: people with stroke.

The evaluation phase of an iterative user-centered design approach supported by a qualitative descriptive methodology was used to address the aims of this study. In this study, users were people who had experienced a stroke and physiotherapists working in stroke rehabilitation. This study consisted of 2 phases. In phase 1, a series of usability testing sprint cycles were conducted [59]. In phase 2, a “near-live” [60] testing approach was used, in which 2 participants with stroke and a physiotherapist undertook a 3-week intervention of locomotor rehabilitation augmented by exciteBCI (Figure 1).

### Ethical Considerations

The study was approved by the New Zealand Health and Disability Ethics Committee (17/NTA/177), and locality authorization was endorsed by the Auckland University of Technology Ethics Committee (17/373). Before the study, all participants provided written informed consent. The privacy and confidentiality of participants was protected by secure storage of all data and deidentification of data where feasible. Participants received an NZ $40 (US $24) gift voucher for each session they attended in acknowledgment of their contributions.

### exciteBCI Prototype

**Figure 2:**
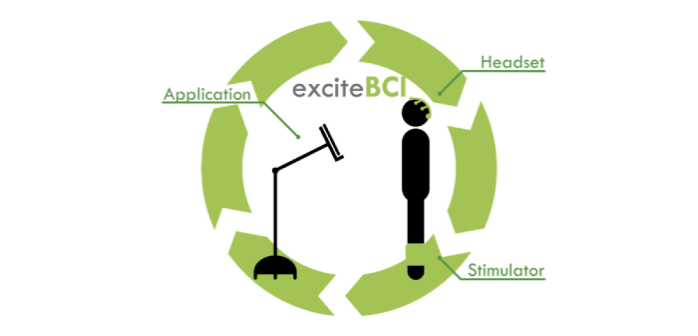
The exciteBCI consists of 3 components: an electroencephalography headset, a muscle stimulator, and a mobile app, each of which communicates wirelessly.

The exciteBCI prototype evaluated in this research is intended for clinical use in collaboration with a qualified physiotherapist in an inpatient, outpatient, or community setting. exciteBCI has 3 components: 2 wearable components, including an electroencephalography headset and a muscle stimulator, and a third component, a mobile app. The 3 components communicate wirelessly (Figure 2).

**Figure 3:**
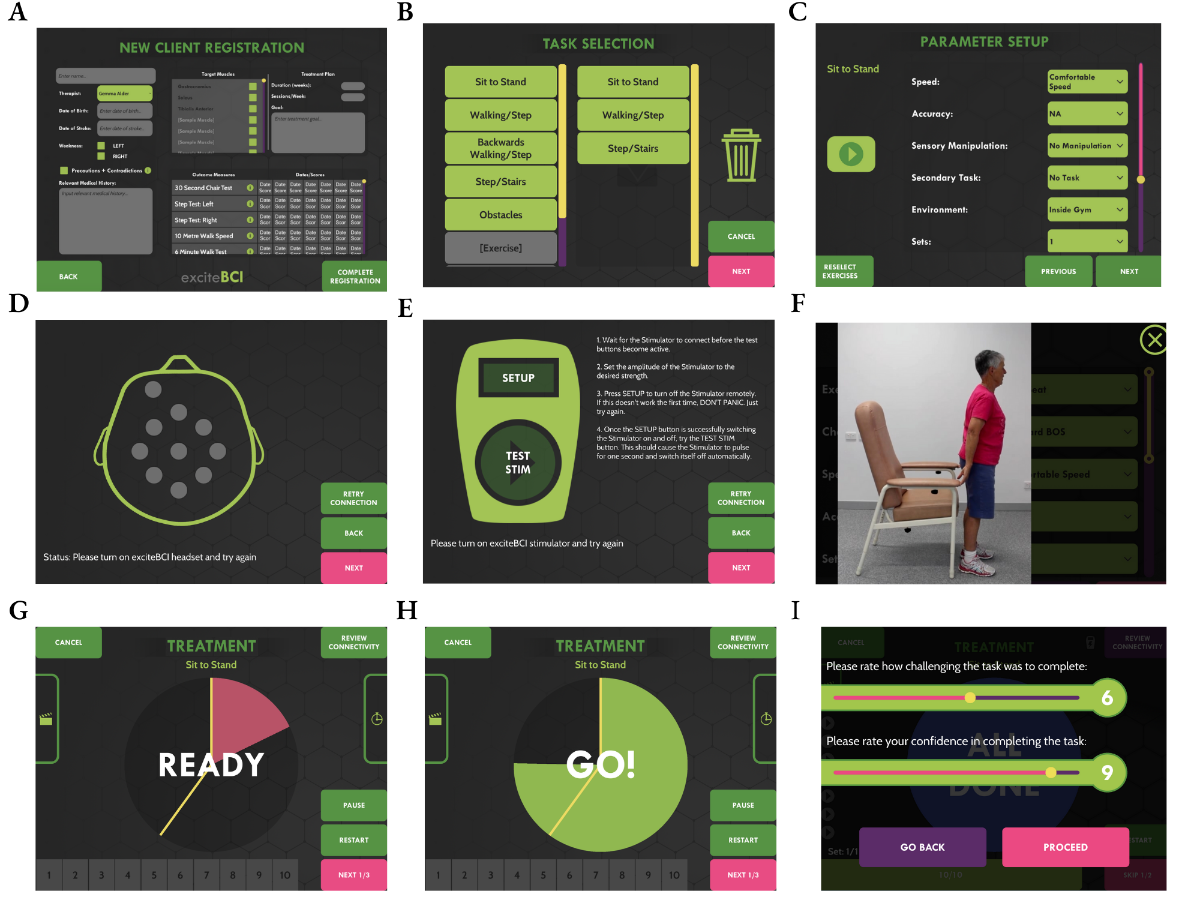
Example screenshots from the exciteBCI app interface prototype v3.3. (A) Registering a new patient, (B) the task selection suite where the patient and therapist select tasks that align with the patient’s goals, (C) the parameters that can be manipulated for each task by the therapist to ensure an optimal level of task difficulty, (D) checking the impedance levels of the electroencephalography headset, (E) the muscle stimulator is connected to the app and stimulation amplitude saved, (F) the patient watches a video on how to perform the task, (G-H) the timing signal (auditory-visual cue) to get ready and execute the task, and (I) the patient completes the task difficulty and confidence ratings at the end of the task set.

The electroencephalography headset included 9 gel electrodes capable of recording brain activity that was used to predict when the person with stroke was going to move. The muscle stimulator (NeuroTrac Rehab) was housed within a neoprene sleeve and worn during rehabilitation tasks to deliver electrical stimulation to a lower limb muscle. The muscle stimulator delivered the afferent stimulus which was paired with electroencephalography brain activity to induce neural plasticity. The exciteBCI app was designed to support the delivery of the intervention. It included locomotor tasks cued with an audiovisual prompt. The locomotor tasks could be selected, and the task parameters such as number of repetitions, movement speed, and rest time manipulated to create an individualized locomotor rehabilitation program. See Figure 3 for example screenshots from the exciteBCI app interface prototype v3.3. This version of the app was presented to participants during the first sprint cycle of phase 1.

### Participants

People diagnosed with a stroke at least 6 months prior who presented with some restriction of the foot and ankle movement limiting locomotor function were recruited. People with English language limitations, cognitive, perceptual, and communication impairments, who were unable to engage in the research process even with the support of a family member or a health professional, were excluded. New Zealand registered physiotherapists with at least 5 years of professional experience in the field of neurological rehabilitation were recruited. Networking with local health care and rehabilitation providers and community advertising were used to recruit a convenience sample of participants. All participants provided written informed consent before participating in the study.

### Procedures and Data Collection

#### Phase 1: Think-Aloud Sprint Cycles

People with stroke (n=4) and physiotherapists (n=4) participated in a single 1-hour usability testing session, where they interacted with the exciteBCI prototype using a “think-aloud” approach [61], followed by a semistructured interview [62] (refer to Figure 1). At the end of each sprint cycle, user device requirements were compiled, and changes were made to the user interface and device before the next sprint cycle began. Participants were asked to use a “think-aloud” process by verbalizing their thoughts, observations, and opinions while interacting with the exciteBCI prototype in a planned series of activities (Textbox 1). The researcher’s interactions were kept to a minimum to support participants to fully engage in the “think-aloud” process, but, when necessary, the researcher prompted the participant with phrases like “tell me what you’re thinking now.” Video and audio recordings, photographs, and researcher observations (TB SO, and UR) were used to capture the think-aloud process. Semistructured interviews were conducted by 2 experienced researchers (GA and NS). The interviews were audio-recorded and focused on participants’ experiences and opinions of the device, functionality, design features, and suggested improvements. See Multimedia Appendix 1 for phase 1 indicative interview questions. Consecutive participants participated in the sprint cycles until no new insights or changes to the exciteBCI device's design features were provided [63,64].

**Examples of activities used in phase 1 sprint cycles to facilitate the “think-aloud” process.**

**Physiotherapists**

- Use the tablet to complete the initial client registration (10-15 min).
- Use the tablet to design a task-specific training program for a client (10-15 min).
- Follow the instructions to set-up the headset (10-15 min).
- Follow the instructions to set-up the electrical stimulation and place electrodes on the tibialis anterior muscle (10-15 min).
- Calibrate the system—complete the task-specific training while the headset records your model’s brain signals (10-20 min).
- Complete task-specific training while your model receives the paired intervention (5-10 min).
- Remove the headset and electrical stimulation device (5-10 min).

**People with stroke**

- Now you are set-up, follow the cue on the tablet to perform the exercises and we will record your brain signals (20-25 min).
- Follow the cue on the tablet screen to complete the exercises while receiving the neuromodulatory intervention (20-30 min).
- Please rate how difficult it was to perform that task and how confident you felt performing the task (5-10 min).
- Donning and doffing of equipment (10-15 min).

As the time spent on each activity varied across participants, a time range (min) has been listed against each activity. Similarly, the number of task-specific training exercises and associated repetitions varied across participants (2-4 exercises 20-80 repetitions per exercise). For the physiotherapist session, the model was a member of the research team.

#### Phase 2: Near-Live Program of Rehabilitation

A “near-live” testing approach [60] was carried out in which 2 people with stroke engaged in a 3-week program of locomotor rehabilitation augmented by exciteBCI (Soft Headset v2, App v3.6, Electrical Stimulator v.3). Eight 1-hour rehabilitation sessions were conducted in an outpatient clinical setting supervised by a New Zealand registered physiotherapist with 10 years of clinical experience in stroke rehabilitation (GA). Before the rehabilitation sessions, participants attended an initial assessment and planning session to establish locomotor-related goals and completed clinical outcome measures. Clinical measures included the 30-second chair stand test, 10-m walk test; 6-minute walk test; four step square test, and lower limb muscle strength testing of the ankle dorsiflexors, ankle plantar flexors, knee extensors, and hip flexors using a handheld dynamometer [65]. Clinical measures were repeated at the end of the rehabilitation program.

The rehabilitation program was based on current evidence-based practice as recommended in the National and International Clinical Guidelines for Stroke [66-68] and included goal-oriented task-specific training of locomotor-related skills that were deemed important to the participant. The physiotherapist prescribed 3 to 4 different tasks from the suite of tasks within the tablet-based exciteBCI app per session. Informed by the principles of motor learning [69], the rehabilitation tasks were progressed over the program based on the participant’s rating of perceived difficulty for each task using a numerical visual analog scale. Task parameters were manipulated or new tasks were prescribed to achieve a challenge point of 6 to 8 out of 10 on the task difficulty visual analog scale for each task [57]. Participants completed between 30 and 100 repetitions of each task during the 1-hour session. The participants used the exciteBCI throughout the rehabilitation program, which delivered electrical stimulation to the tibialis anterior muscle to coincide with the person’s intended movement. Approximately 10 minutes of the 1-hour session was attributed to the donning and doffing of the equipment.

All rehabilitation sessions were video-recorded, researcher observation notes (NS and TB) of the participant interactions with the exciteBCI prototype were recorded, and photographs were taken at each session. Following completion of the rehabilitation program, the participants with stroke and the physiotherapist (GA) took part in separate semistructured interviews. Interviews were conducted by 2 experienced researchers (GT and SM) who were not involved in the development of the exciteBCI. The interview focused on the participants’ opinions and experiences of using the device within a rehabilitation context and included specific questions in response to video observations of the rehabilitation program. All interviews were audio-recorded. See the Multimedia Appendix 1 section for phase 2 indicative interview questions.

### Data Analysis

“Think-aloud” and interview data were transcribed verbatim. The transcripts and written observation notes were imported into NVivo 12 (Lumivero) computer software package [70]. Data analysis was undertaken in 2 stages. In the first stage, transcripts and videos were descriptively analyzed by the primary researcher (GA) to identify user interface and device requirements within each sprint cycle. This analysis was then discussed with the team to inform the development of the device before the next cycle. This analysis also served as a familiarization process for the second stage of analysis.

The second stage of analysis focused on addressing the aim, exploring users’ perspectives and experiences of exciteBCI, and how well it fits with rehabilitation. This stage used a modified version of conventional content analysis [71] to analyze the data from both phases of the study. Conventional content analysis allows the researcher to immerse themselves in the data to acquire an accurate description of what participants experienced and understood about the topic at hand [62]. The data were coded inductively by the primary researcher (GA) at the sentence or phrase level, and a semantic coding framework was iteratively developed during the data analysis process [72]. Several activities were used to enhance the understanding of code relationships, such as continuous comparisons within and between codes and data sources, as well as the practice of memoing to capture initial insights about the data and potential interactions among codes [73]. The coded data iteratively informed the development of categories. Categories and representative coded data were visually represented using a mind map in the MIRO Application to support the development of themes. The coded data, categories, and prototype themes were reviewed and discussed with 2 researchers (NS and GT) in a series of analysis meetings to ensure consistency of interpretation.

### Results

#### Overall

**Figure 4:**
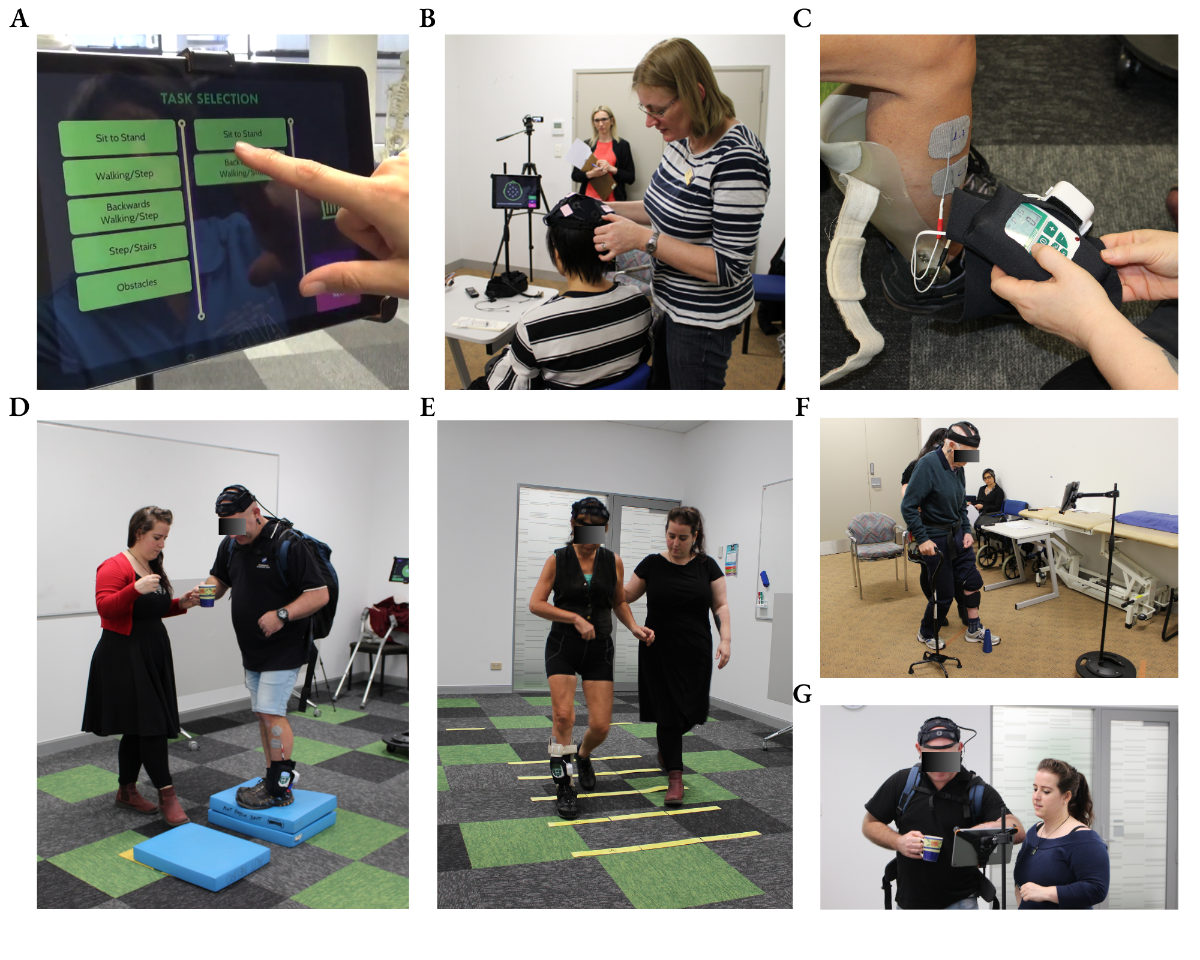
Photographs of participants from phase 1 sprint cycles and phase 2 “near-live” 3-week program interacting with the exciteBCI device. (A) A participant with stroke and physiotherapist work in partnership to select task-specific training exercises in the exciteBCI app, (B) a physiotherapist participant setting up the electroencephalography headset, (C) a physiotherapist setting up the muscle stimulator on a participant with stroke, (D-F) participants with stroke engaging in locomotor rehabilitation while wearing the exciteBCI device and receiving the neuromodulatory intervention with the physiotherapist, (G) a participant with stroke using the app rating scale of perceived rehabilitation task difficulty to inform the physiotherapist about the challenge-point of the task.

A total of 11 people participated in the study with no reported adverse events. Table 1 presents the demographic and clinical characteristics of the participants, and Figure 4 displays photographs of participants interacting with the device in phases 1 and 2 of usability testing. In the interest of intellectual property protection, the iterative device requirements for the user interface and the device are not presented in this paper.

**Table 1:** Participant characteristics (n=11).

Demographic and clinical characteristics					Value
**People with stroke (n=6)**					
	**Type of stroke, n (%)**				
		Ischemic			3 (50)
		Hemorrhagic			3 (50)
	**Lesion location, n (%)**				
		Right hemisphere			4 (67)
		Left hemisphere			2 (33)
	**Time since stroke (y), mean (SD; range)**				7 (6.7; 2-19)
	**Types of impairments, n (%)^a^**				
		Motor			6 (100)
		Sensory			4 (67)
		Perceptual			2 (33)
		Cognition			2 (33)
		Communication			1 (17)
	**Functional ambulation category scores [74], n (%)**				
		0^b^			0 (0)
		1^c^			0 (0)
		2^d^			1 (17)
		3^e^			1 (17)
		4^f^			3 (50)
		5^g^			1 (17)
	**Prior experience of technology components, n (%)**				
		**FES^h^**			
			Clinical	4 (67)	
			Research	3 (50)	
			No experience	1 (17)	
		**BCI^i^**			
			Clinical	0 (0)	
			Research	3 (50)	
			No experience	3 (50)	
		**Mobile app–based interventions**			
			Clinical	0 (0)	
			Research	0 (0)	
			No experience	6 (100)	
	**Sex, n (%)**				
		Female			3 (50)
		Male			3 (50)
	**Ethnicity, n (%)**				
		Asian			1 (17)
		European			2 (33)
		New Zealander			3 (50)
	**Age (y), n (%)**				
		<45			1 (17)
		45-65			2 (33)
		>65			3 (50)
**Physiotherapists (n=5), n (%)**					
	**Years qualified as a physiotherapist**				
		5-10			1 (20)
		10-20			3 (60)
		>20			1 (20)
	**Highest qualification**				
		Bachelor of Science			4 (80)
		Masters			1 (20)
	**Years of clinical experience in stroke rehabilitation**				
		5-10			3 (60)
		10-20			1 (20)
		>20			1 (20)
	**Prior experience of technology components**				
		**FES^j^**			
			2-12	5 (100)	
		**BCI^k^**			
			3 (research context)	1 (20)^l^	
		**Mobile apps for rehabilitation^m^**			
			3-10	5 (100)	
	**Sex**				
		Female		5 (100)	
	**Ethnicity**				
		European			2 (40)
		New Zealander			3 (60)

^a^n>6 due to some participants presenting with multiple impairments.

^b^Nonfunctional walker (unable to walk).

^c^Dependent walker requires continues manual contact.

^d^Dependent walker requires intermittent manual contact.

^e^Dependent walker requires verbal supervision or guiding.

^f^Independent walker on level surfaces only.

^g^Independent walker on any surface.

^h^FES: functional electrical stimulation (n>6 due to experience in more than 1 category).

^i^BCI: brain computer interface.

^j^Years experience using functional electrical stimulation.

^k^Years experience using brain computer interfaces.

^l^Primary author: GA.

^m^Years experience using mobile apps for rehabilitation. Video mobile apps to capture patient performance and exercise provision mobile apps.

**Figure 5:**
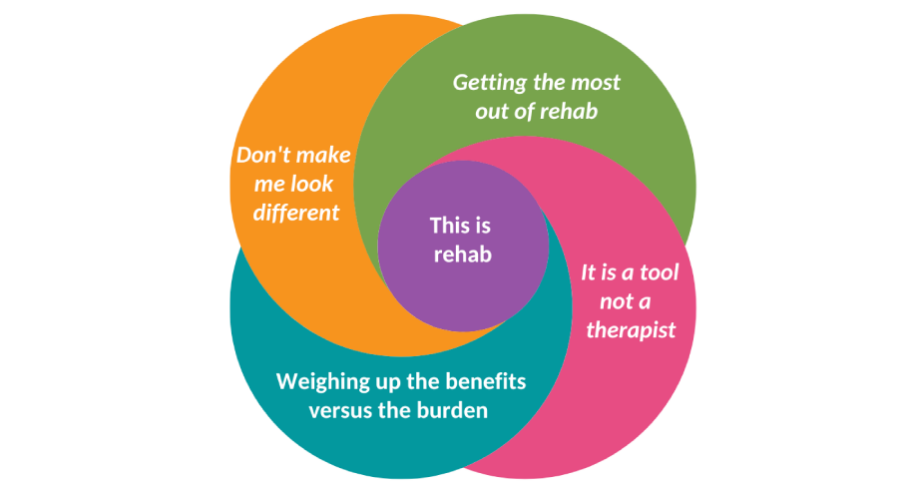
The relationship of themes associated with the users’ perceptions and experiences of exciteBCI, and how well it fits with rehabilitation.

Overall, the findings showed that participants with stroke and physiotherapists had positive perceptions and experiences of the exciteBCI intervention and could see it being used in a rehabilitation context. Five themes were generated from the data, as illustrated in Figure 5. Central was the theme (1) This is rehab, which interacted and was influenced by the themes (2) Getting the most out of rehab, (3) It is a tool not a therapist, (4) Weighing up the benefits versus the burden, and (5) Don’t make me look different. Illustrative quotes that corroborated the data have been selected for thematic representation, and pseudonyms have been used.

#### Theme 1: This Is Rehab

At the core of the findings was the theme, “This is Rehab.” Despite being a novel technology incorporating a brain computer interface and app, both physiotherapists and people with stroke identified that the exciteBCI was clearly a rehabilitative tool suitable for supporting and augmenting rehabilitation practice:

At first, I thought, it’s not a new treatment [...] we’re doing something that’s extra, but it’s basically just facilitating what we’re doing anyway.

Sarah, Physiotherapist, Sprint cycle 2

For clinical use, the intervention worked brilliantly.

Anni, Age 58, PwS, 3-wk program

Many expressed surprise at how congruent the technology was with their own clinical practice and understanding of rehabilitation principles. This congruence appeared to enhance perceptions of usability and acceptability. They readily identified ways in which the technology could be integrated into current rehabilitation practices:

It doesn’t seem to be over complicated, and once you use it on a daily basis more or less it’s actually not that difficult.

Zoe Physiotherapist, Sprint cycle 2

Participants were easily able to understand the purpose and mechanism of the intervention. Physiotherapist participants drew on their understanding of functional electrical stimulation (FES), a modality commonly used in clinical practice, in understanding the physiological underpinnings of the paired neuromodulatory intervention. They identified that the exciteBCI device could offer benefits over FES, particularly in relation to the way the person’s own brain signals are used to drive the delivery of electrical simulation, and suggested that this would enhance outcomes from rehabilitation:

With an external stimulus like the FES hand switch, I need to try and time it right. So, it’s not that it’s not inconvenient it’s just[...] makes more sense to take the relationship internally rather than externally. It’s the patients driving it[...] And I think that will facilitate learning more.

Sarah, Physiotherapist, Sprint cycle 2

Physiotherapists described how the exciteBCI app would support them in delivering effective rehabilitation by supporting their clinical reasoning process, enabling specificity in the design of rehabilitation programs, and promoting efficiency. They particularly valued how the exciteBCI app aided them in thinking about different aspects of task-specific training. However, they also consistently emphasized the importance of tailoring rehabilitation to individuals.

This prompted them to describe additional app features which might support further personalization:

What this shows [points to video playing task], it’s the goal of doing it perfectly well, but it might not look like that for them [the client]. Would be great if the actual client performing the task can be videotaped as well.

Sarah, Physiotherapist Sprint cycle 2

Shopping bags, washing basket so they are the things that we would [...] quite commonly do, but [...] when I choose secondary tasks, I often choose things that I know this patient is going to do and relates to their goals [...] so a customize option is essential

Zoe, Physiotherapist Sprint cycle 2

Attention to an efficient workflow, minimizing duplication of information being entered, and system interoperability were also priorities for physiotherapists. They particularly valued how the exciteBCI app could support efficient and comprehensive clinical record keeping and support handover between therapists:

I would think about a lot of these different parameters, but I might not be as explicit about them in my notetaking [...] this is way more thorough[...] structured[...] easy to follow, easy to pick up on for next time[...] and so much quicker.

Megan, Physiotherapist, Sprint cycle 1

Physiotherapists identified the challenges associated with the use of clinical terminology and language, calling for app features such as icons and pop-up definitions, which would support users to have a clear understanding of key terms:

Accuracy? so I’ve got large target, small target, wide path, small path [...] so path I presume means width? [...] I think photos or icons is a really good thing to just clarify stuff- probably more than words.

Megan, Physiotherapist, Sprint cycle 1

Most physiotherapists also described the need for prior hands-on training opportunities led by an experienced clinical expert, specifically addressing the rehabilitation approach, device set-up, and troubleshooting. This was deemed essential to support the successful uptake of the technology in rehabilitation. In addition, they recommended incorporating training and troubleshooting videos directly into the app.

#### Theme 2: Getting the Most Out of Rehab

People with stroke and physiotherapists described the ways in which the exciteBCI, and in particular the app, could support people with stroke to engage in more intensive and challenging rehabilitation at higher doses. Participants valued the way exciteBCI supported people with stroke to work hard. This was achieved in multiple ways. First, the app enabled physiotherapists to design specific and challenging rehabilitation programs for patients:

It just was a really nice, structured way of [...] finding that sweet spot to maintain challenge, but for the participants to feel they are making progress and all that hard work is paying off.

Gemma, Physiotherapist, 3-wk program

Second, the task difficulty rating scales supported both people with stroke and physiotherapists to judge the challenge of each task and served as a prompt to work on more challenging tasks:

[...]and the rating scale is really helpful too. You have a bit more about what’s challenging for them and what’s not. Gives you a better starting point.

Zoe, Physiotherapist, Sprint cycle 2

Third, the audiovisual cue that prompted the onset of movement compelled people with stroke to remain focused. By setting the time of task practice, limiting the amount of rest, and promoting attention on the task, large volumes of rehabilitation were achieved:

[...]I think the way that the system has been constructed is quite focus oriented...It’s actually keeping the time [...] you get so much more in [the session].

Anni, Age 58, PwS, 3-wk program

Together, these factors supported people with stroke to work at intensities and volumes beyond what they would normally achieve during rehabilitation. This supported substantial gains in balance and walking and, in turn, built self-efficacy:

I felt a sense of achievement you know, and that’s what’s important, I’m still making progress.

Jake, Age 44, PwS, 3-wk program

These findings illustrate that both people with stroke and physiotherapists discovered a number of features within the app that support fundamental rehabilitation principles.

#### Theme 3: It Is a Tool, Not a Therapist

Participants with stroke emphasized that developing a trusted relationship with their therapist was fundamental to their rehabilitative journey. They discussed ways in which the app might support or disrupt this relationship. Participants in phase 1 raised the possibility that the app could disrupt the therapeutic relationship:

[...]Physically having the device and therapist there complicates the relationship...so the therapist really needs to make sure their cues show engagement and interest[...] there is a risk here.

Thonia, Lived experience researcher

While those who participated in the 3-week program did not highlight the same concern, all participants stressed that the app was a therapeutic tool that should not be viewed as a substitute for the therapist:

[...] if you feel I handed over the therapy session to this [app] and I've cognitively left the building, then that is disastrous, to our relationship and their treatment. If I'm not engaged, why would they want to be

Jude, Physiotherapist, Sprint cycle 3

Most participants indicated that they would be happy to use the app independently; however, the majority indicated a preference for using the device with the physiotherapist present. They described how the physiotherapist offered guidance and feedback about their performance and progress:

So, if I'm not doing a sit to stand correctly [points at app] then who is going to correct me? [...] I like the feedback from the therapist, because at some point the quality of the movement does matter.

Lilly, Age 64, PwS, Sprint cycle 1

Participants also described how the physiotherapist motivated them to work harder by encouraging them to do their best work, while also understanding their personal limits in a way that the app could not:

The physiotherapist can push you further. They can see that you can be pushed extra. Which is important [...] people get tired, aagh and ready to give up, whereas the therapist goes another 10-minutes. Helps you squeeze out that last little bit.

Bob, Age 84, PwS, Sprint cycle 1

Consequently, some participants felt that the exciteBCI either should not or could not be used without the support of a physiotherapist. These findings illustrate that attention to the impact of technology on the therapeutic relationship during both the design and implementation of rehabilitation technologies is essential.

#### Theme 4: Weighing up the Benefits Versus the Burden

While both people with stroke and physiotherapist participants saw the benefits and potential of exciteBCI, they also called for “real-world evidence” of effect, research-based evidence, or endorsement from a trusted source:

[...] If I knew it was going to bring about a speedier recovery, I would be more likely to use it.

James, Age 74, PwS, Sprint cycle 2

If my physio turned up with it, I wouldn’t mind at all.

Jenny, Age 67, PwS, Sprint cycle 2

Those who participated in the 3-week program drew on their own experiences of the intervention to generate real-world evidence:

When we visited French festival, I was helped up onto the raised platform advertising electric cars [...] Renault Twizy. I don’t think I was pressing down on my husband’s hand as much, on ‘reaching’ up, and managed to squeeze myself into the car-space for a photo or two, [...] great! Extending my reach, even just a wee bit, was brilliant.

Anni, Age 58, PwS, 3-wk program

Physiotherapists described how they would consider cost, client suitability, set-up time, workflow, and clinical effectiveness when deciding whether to adopt the exciteBCI intervention:

I would need to know it was going to make a really big difference to invest in purchasing it and have a fair amount of clients I could use it with. [...] it’s difficult without actually using it in clinical practice as to know sort of who would really benefit.

Megan, Physiotherapist, Sprint cycle 1

While participants with stroke called for the device to be integrated into their rehabilitation as early after stroke as possible, some physiotherapists saw a tension between the set-up time of both the headset and the app and the decision to implement the device in different clinical contexts:

I don’t think[…] you’d use it on an acute ward[...] I think it takes too much time [...] based on how much time[...] physios have based in my experience[...] I just think time and all the equipment there and everything else that goes on. I don’t, can’t see it working. In a rehab ward maybe. Definitely outpatients.

Jude, Physiotherapist, Sprint cycle 3

When discussing use of the device in the home, participants with stroke and physiotherapists from phase 1 (single session) viewed the headset as a potential barrier to adopting the device. This was mainly due to the perceived difficulty of setting it up independently for those with upper limb disability and the need for gel to be inserted into the electroencephalography electrodes while wearing the headset. The set-up of the electrical stimulator and app was not viewed in the same light. While people with stroke who participated in the 3-week program expressed similar concerns, they were eager to offer suggestions to make it a viable option for independent use.

#### Theme 5: Do Not Make Me Look Different

Almost all participants emphasized how the social acceptability of the device would influence their desire to use it. The need for a socially acceptable device design was less important in a clinical setting or at home than if the device was being used in a public or social context:

It’s [the headset] not for glamour it’s for results [...] it’s the job it’s doing, reading the brain. Glamour doesn't matter, does it? If there’s certain areas of the brain it can read, it’s got to work. It doesn’t matter what it looks like [...] not whilst you’re doing rehab. You’re there for the rehab.

Lilly, Age 64, PwS, Sprint cycle 1

Nevertheless, most participants called for a device which did not draw attention to themselves or their disabilities:

Many people find it difficult to approach you, when you walk differently, or they treat you differently [...] So, you can understand why I would value a design that doesn't make me look even more different than I already do.

Jenny, 67 years; PwS, Sprint cycle 2

Participants posited that what constitutes a socially acceptable device might also vary depending on a person’s gender, age, and culture. The length of time since their stroke diagnosis and how much rehabilitation was prioritized in their daily lives also appeared to influence the participants’ perspectives on whether they would consider using the wearable device in their daily lives:

Initially I would [during inpatient rehab] [...] you’re ready to take anything that you think will help but what shifts the balance of that is I think it’s about - I’m more progressed now and more - I want people to see me where I’m at now.

James, Age 74, PwS, Sprint cycle 2

Concerns about the social acceptability of the device largely pertained to the esthetics of the headset and electroencephalography gel. The current headset design was viewed as unacceptable for wearing-out in public, where social perceptions play a role. Minimizing and concealing the device with clothing or incorporating it into something that looked familiar and fits with everyday life, such as a hat or headphones, was seen as an important future design consideration. Participants from phase 1 perceived there to be no issue with the use of electroencephalography gel in a rehabilitation environment. While participants in the 3-week program described the inconvenience of repeated gel use and its impact outside of the rehabilitation context, they perceived that the benefits they experienced from the intervention outweighed this inconvenience. In contrast, participants perceived the electrical stimulator to be more acceptable. It was noted that the electrical stimulator and its neoprene housing resembled sports braces which were considered socially acceptable and in common use.

### Discussion

#### Principal Findings

This study applied a user-centered design approach to explore the perspectives and experiences of people with stroke and physiotherapists when engaging with exciteBCI rehabilitation technology and how well it fits within a stroke rehabilitation context. The results support the acceptability of the exciteBCI intervention and its “fit” with clinical practice and will inform the requirements for future device development. These findings also provide key insights that can inform the design and implementation of rehabilitation technologies more broadly.

#### Technology and the Therapeutic Relationship

This study highlights the importance of considering the impact of technology on the therapeutic relationship between patients and therapists. The therapeutic relationship refers to the relational process that takes place during clinical interactions and is considered a critical aspect of rehabilitation by both patients and therapists [75-77]. Qualitative research in neurorehabilitation indicates that positive therapeutic relationships are pivotal in supporting patient engagement, positive patient experiences, and in enhancing patient outcomes [78,79]. The “It is a tool, not a therapist” theme identified that both people with stroke and physiotherapists who participated in the phase 1 single usability sessions cautioned that the exciteBCI app could be disruptive to the therapeutic relationship. Similar concerns have been raised in other health technology domains, citing disruptions of the therapeutic relationship as a potential barrier to technology adoption [80-83]. In contrast, in this study, participants who used the exciteBCI app over multiple sessions in phase 2 highlighted how the technology could be successfully integrated into clinical interactions to support and enhance the therapeutic relationship. The participants described how using the rating scales within the exciteBCI app facilitated a shared understanding of the rehabilitative challenge, allowing the person with stroke to take control of their rehabilitation and identify opportunities for progressing rehabilitation challenge in collaboration with the physiotherapist. However, this required the physiotherapist and the person with stroke to be mindful of the role of the technology, its value, and limitations, and to use it purposefully and appropriately.

The current findings combined with previous literature emphasize how crucial it is to take the patient, therapist, and technology triad into account when designing and implementing rehabilitation technology. While research in telehealth has attended to the influence of technology on the therapeutic relationship over time and its implications for usability [84-86], the same attention has not been given to the development of rehabilitation technology devices. Understanding the influence of technology on clinical interactions and workflow, and the ways in which these change over time, is critical. Being attuned to the impact rehabilitation technologies have on therapeutic relationships requires a deep understanding of the role of the therapist and patient and the rationale for the technology. This knowledge should be applied in two ways: (1) designers and developers should explicitly consider how the design of the technology can support and strengthen the therapeutic relationship, and (2) therapists and health care educators should consider how rehabilitation technologies can be used to support person-centered rehabilitation, ensuring that the therapeutic relationship is preserved and developed throughout the rehabilitation process.

#### Considerations for Social Acceptability in Rehabilitation Technology Design

The findings from the “Don’t make me look different” theme clearly articulate that people with stroke place weight on the esthetics and social acceptability of a rehabilitation technology device when considering whether to use it. Social acceptability may be particularly important in the design of head-mounted wearable devices [87-89]. This is an important finding given that social acceptability is often a lesser priority in the rehabilitation technology development process. Defining the target user population and understanding their device requirements early in the design and development process may resolve acceptability issues and minimize the need for significant design changes later in the process [90]. This approach has the potential to not only mitigate issues of acceptability and usability but also reduce the impact on development time and costs, while simultaneously increasing the chances of successful adoption and sustained use of the technology [45,91]. Importantly, our findings indicate that the need for a socially acceptable device design is dependent on the context in which the technology is to be used. Therefore, device developers should be cognizant of the use context when prioritizing social acceptability. Given the shift in rehabilitation services to the community [92] and supported self-management programs [93], social acceptability for both users and bystanders [87,89] is likely to be a pivotal consideration in the design of rehabilitation technologies in the future.

#### User Testing in a Sustained Way

User-centered design approaches are increasingly being encouraged to inform health technology design and development [45,94]. Yet much of the methodological literature describes single-session usability evaluation, where novice users’ perceptions and experiences of the technology inform the next iteration [95]. While this approach can highlight “entry-level” usability issues or novice user frustrations that can often be quick fixes, it is unlikely to identify fundamental usability and acceptability issues or the sources of frustrations that could be barriers to the sustained use of the technology [59,95]. This is an important limitation when using user-centered design methods for the development of rehabilitation technologies intended for sustained use. To ensure that we captured design requirements that support both the adoption and sustained use of the exciteBCI technology, in addition to the iterative single usability evaluation sessions, we conducted “near-live” usability testing over a 3-week period. This approach allowed us to investigate the technology’s “fit” with a program of rehabilitation. Users’ long-term experiences and the ways in which the device’s usability and acceptability evolved over time were elucidated.

An important finding was that user perspectives and usability priorities shifted with sustained use of the exciteBCI technology. Long-term users were less concerned with the practicalities of the technology, such as the ease of setting up the exciteBCI app and headset. While these inconveniences were noted, they were apparently offset by the benefits users experienced from the intervention itself. It appeared that from engaging in sustained use, they obtained the “real-world evidence” in support of the technology which was desired by participants in phase 1. Long-term users also did not highlight that the technology might disrupt therapeutic relationships. Instead, they were more focused on the pleasure they had from “getting the most out of rehab,” the increase in self-efficacy they experienced, and the gains they made when the technology augmented the rehabilitation process. Similar findings have been previously reported in the literature. When meaningful connections are formed with technology over time, the pleasure derived from its use acquires more weight, and practical limitations become less significant [96]. A single-session usability assessment may not always foresee future satisfaction with the technology since it may be evaluating expectations rather than user experience [97]. In the context of this study, it is critical to remember that the user experience includes not only the technology itself, but also the experience gained from participating in the rehabilitation process, and that this experience occurs within the context of a therapeutic relationship. As a result, explicitly designing technology to support both meaningful rehabilitation and best practice from the physiotherapist to establish and nurture a therapeutic relationship is paramount.

Our findings regarding the sustained use of exciteBCI technology also provide important guidance for its eventual implementation in clinical practice. Current models for implementing rehabilitation technologies often rely on instructional booklets and training and accreditation packages, usually provided by the technology manufacturer. While these strategies are likely to support the adoption of rehabilitation technology, they are less likely to support sustained use. Implementation science literature emphasizes the importance of strategies, such as identifying factors and barriers to sustained use, the use of clinical champions on site, and the use of behavior change strategies to ensure therapists are supported in practice change [98-100].

#### Limitations of the Study

This study adopted a rigorous approach to qualitative research; however, it is important to acknowledge that we used a convenience sampling method in this study; all participants were in the chronic stage of stroke (2-19 y since stroke onset), and the physiotherapists worked in an outpatient rehabilitation or community setting. While participants reflected on their prior lived experiences of inpatient rehabilitation closer to the onset of their stroke, when their stroke symptoms were more severe, or when physiotherapists worked in an inpatient setting, future user testing should include participants who are currently undergoing or providing inpatient rehabilitation. This will determine whether their experiences align with or differ from the device features described in this study and help to understand how well it fits in an inpatient rehabilitation setting. In phase 1, while the physiotherapists had extensive experience with the patient cohort and their associated clinical presentation, the model used during the usability evaluation was not an individual with stroke. This may have influenced the usability results. Only 1 physiotherapist (GA) participated in the “near-live” 3-week program. Future research should explore usability testing over periods of time with a range of different users. Another potential limitation of the study design was that it did not capture the initial expectations of phase 2 long-term users. Therefore, we were unable to interpret how their initial expectations may have shaped their experiences of using exciteBCI, and whether their expectations were confirmed or disregarded at the end of the program.

#### Conclusions

This study presented an iterative user-centered design approach supported by a qualitative descriptive methodology exploring users’ perspectives and experiences of exciteBCI, a complex neuromodulatory rehabilitative technology designed to augment locomotor rehabilitation for people with stroke. The 5 interrelated themes generated from the analysis revealed that overall exciteBCI was perceived and experienced positively by people with stroke and physiotherapists and viewed as technology that could be implemented in a rehabilitation context. These findings provide important insights pertinent to the broader field of rehabilitation technology design, implementation, and sustained use. Notably, these findings highlight that rehabilitation technology design should (1) consider ways to support and enhance the therapeutic relationship; (2) recognize that social acceptability is a design imperative, but its significance varies depending on the use context; and (3) that there is merit in using research design methods that explore device usability within the context of sustained use.

## Appendix

Multimedia Appendix 1 Indicative interview questions.

## Abbreviations

FES: functional electrical stimulation
ISO: International Organization for Standardization

## Data Availability

The data sets generated and analyzed during this study are not publicly available because of ethical approval requirements, which do not permit data sharing. Data may be obtained from the corresponding author upon reasonable request with the approval of the relevant ethics committee.
