# Supplementary material for: A Brain Computer Interface Neuromodulatory Device for Stroke Rehabilitation: Iterative User-Centered Design Approach
Source: JMIR Rehabil Assist Technol. 2023 Dec 11;10:e49702. doi: 10.2196/49702 (PMC10750233; doi:10.2196/49702)
Supplement: Multimedia Appendix 1 [file rehab_v10i1e49702_app1.docx]

**Supplementary Material**

***Phase one sprint cycles: Semi-structured interview framework***

- Can you describe your thoughts about the exciteBCI? likes/dislikes
- Can you please tell me about… (Mobile App, Headset, Stimulator)
- Aesthetics
- Comfort and fit
- Support
- Suitability – during exercise
- Content
- Ability to follow onscreen instructions
- Screen readability
- Understandability
- Linking devices effectiveness
- Workflow
- Practicality
- Physical issues
- What improvements would you suggest?
- Was there anything you were looking for on the device or the App that you couldn’t find?
- Can you describe how easy the exciteBCI and app have been to use from your perspective?
- How would you envisage the exciteBCI being used?
- Who do you think the exciteBCI would be suitable for?
- What contexts (inpatient, outpatient, residential care, community, home, rural settings) would you imagine using this device?
- Would a person using the exciteBCI need a support person to set-up and use the device, or could they manage it independently?
- How well does the exciteBCI align with your thoughts on rehabilitation?

***Phase two ‘near-live’ programme of rehabilitation: Semi-structured interview frameworks***

**Framework for Participants with Stroke**

*“I understand that you have been involved in a research project that is looking at the usability and acceptability of a device called exciteBCI as part of a 3-week rehabilitation programme. I would like to ask you some questions, discuss your personal experience and perceptions of using the exciteBCI device. The purpose of this interview is to understand what people with stroke think about exciteBCI and how it might be improved in preparation for translation into rehabilitation”.*

**General:**

- Can you tell me about your overall impressions of exciteBCI?
- What have you liked / disliked about your experience using exciteBCI?

(Suitability of the exercises, support, effectiveness, using technology, therapist, ability to follow app instructions, the visual and auditory cue, the rating scales, physical issues, electrical stimulation sensation…)

- How was the exciteBCI device and programme similar or different from your past experiences of rehabilitation?
- I understand that before you started the treatment sessions you discussed with your physiotherapist (Gemma) what you wanted the rehabilitation sessions to focus on. How well did the sessions meet your needs/ expectations?
- Often rehabilitation involves a person with stroke working directly with a physiotherapist, with not a lot of equipment or gadgets. What was it like to have a device, mobile app and a cue used in rehabilitation?

(Supports or hinders engagement / ability to be seen as an individual /perception of therapist ‘expertise….)

- How has the exciteBCI rehabilitation programme impacted /affected you?
- If positive, what was it about the exciteBCI device and programme that made it positive? (Why does it work?)
- If negative, what was it about the exciteBCI device and programme that made it negative? (Why doesn’t it work?)

**What exciteBCI does and its components:**

*“I would like to focus a little more on the actual device and its different components. “I understand there are three main components a headset, a muscle electrical stimulator and an exercise mobile app”*

- Can you tell me a bit about your understanding of how the exciteBCI device works?

(Components – headset, app with personalised exercises, it pairs the brain (EEG) signal with the muscle stimulation to strengthen the pathways to the muscle)

**Headset**

- Can you please tell me about what it was like setting up and wearing the headset?

(Tell me about…Comfort, fit, weight, suitability during exercises, the use of the gel on your head, dealing with dried gel in the hair post sessions was this a burden or not?)

- Do you have any suggestions on how the headset could be improved?

**Electrical stimulator unit, electrodes, neoprene sleeve**

- Can you please tell me about your experience of using the muscle electrical stimulator on your leg muscle?

(Tell me about…comfort, fit, weight, suitability during exercises, stimulus sensation during set-up and during exercise could they feel it, was it distracting)

- Do you have any suggestions on how the electrical stimulator could be improved?

**MobileApp**

- Can you please tell me about what it was like using the exercise app?

(Tell me about…, screen readability, ability to follow onscreen instructions, exercise availability)

**Visual Auditory cue: (show picture)**

- Tell me what it was like to follow the ready/ go cue?

(Timing movement with the cue challenging or not. If that depends, what did it depend on?)

- Did you tend to pay more attention to the sound of the cue, the visual representation or both?
- Why do you think you were more focused on the sound / visual part of the cue?
- What was it like following the cue on the app and performing the exercises at the same time?
- Do you have any suggestions on how the cue could be improved?

**Rating scales: (show picture)**

*“I understand that once you completed a set of exercises you were asked to rate the exercises”.*

- Can you tell me about your experience using these rating scales?

(Ease of use, confusion around meaning?)

- What were you considering when you were rating difficulty of the task?

(Did it relate to just the physical task or concentrating on the task/ cognitive component)

- What were you considering when you were rating your confidence to perform the task?

(Whether you completed it, how it made you feel pleased, nervous etc.)

- How did it feel rating the exercise after completing every set of reps?
- Do you have any suggestions on how the rating scales could be improved?

**Technical difficulties with device**

- I understand that there were some technical difficulties with the device during your sessions. How did this impact your experience?
- Did these technical difficulties impact the ability of the therapist to carry out your rehabilitation programme?

**Suitability**

- Who do you think the exciteBCI would be suitable for?
- Who is the exciteBCI not suitable for?
- Would a person using the exciteBCI need someone assisting or supervising to set-up and use the device, or could they manage it independently? How could this be improved?
- How long did the exciteBCI system take to set-up? Was this acceptable to you?

**Future use**

- Would you consider using the exciteBCI device in the future? If so, why? If not, why not?

*“I don’t have any further questions is there anything else you would to tell me about your experience?”*

**Framework for Physiotherapy Participants**

*“I understand that you have been involved in a research project that is looking at the usability and acceptability of a device called exciteBCI as part of a 3-week physiotherapy rehabilitation programme”. I would like to ask you some questions, discuss your personal experience and perceptions of using the exciteBCI device. The purpose of this interview is to understand what therapists think about exciteBCI and how it might be improved in preparation for translation into rehabilitation”.*

**General:**

- Can you tell me about your overall impressions of exciteBCI?
- What have you liked / disliked about your experience using exciteBCI?

(Ability to follow app instructions, the visual and auditory cue and the rating scales, physical issues, suitability of exercises, support, effectiveness)

- How was the exciteBCI device and programme similar or different from your past experiences of your rehabilitation practice?
- How well did the exciteBCI meet your needs/ expectations of a rehabilitation technology?
- Often rehabilitation involves a person with stroke working directly with a physiotherapist, with not a lot of equipment or gadgets. What was it like to have a device, mobile app and a cue used in rehabilitation?

(Supports or hinders engagement / ability to be seen as an individual / therapeutic relationship /perception of therapist ‘expertise….)

- How do you think the exciteBCI affected the participants?

- Did the effect of the programme vary? Did some participants benefit more or less?

- If positive, what was it about the exciteBCI device and programme that made it positive? (Why does it work?)

- If negative, what was it about the exciteBCI device and programme that made it negative? (Why doesn’t it work?)

o Do you think that the effort that the participants put into the programme was worth the benefits they got out of it? Why was it worth the effort? Why wasn’t it worth the effort?

o Are there any ideas or concepts from the rehabilitation programme you will integrate into your clinical practice?

**What the exciteBCI device does and its components**

*“I would like to focus a little more on the actual device and its different components. “I understand there are three main components a headset, a muscle electrical stimulator and an exercise mobile app”*

- Can you tell me a bit about your understanding of how the exciteBCI device works?

(Components – headset, app with personalised exercises, it pairs the brain (EEG) signal with the muscle stimulation to strengthen the pathways to the muscle)

**Headset**

- Can you please tell me about what it was like setting up the headset?

(Tell me about…Comfort, fit, weight, suitability during exercises, the use of the GEL, connectivity?)

- Do you have any suggestions on how the headset could be improved?

**Electrical stimulator unit, electrodes, neoprene sleeve**

- Can you please tell me about your experience of using the muscle electrical stimulator during rehab?
- Do you have any suggestions on how the electrical stimulator could be improved?

**App**

o Can you please tell me about what it was like using the exercise app?

(Tell me about screen readability, ability to follow on screen instruction, exercise availability, training parameters, customisability etc…)

**Visual Auditory cue: (show picture)**

o Tell me what it was like to use the ready/ go cue?

o Did patients tend to pay more attention to the sound of the cue, the visual representation or both?

o Why do you think they were more focused on the sound / visual part of the cue?

o Do you have any suggestions on how the cue could be improved?

**Rating scales: (show picture)**

*“I understand that once they completed a set of exercises participants were asked to rate the exercises”.*

o Can you tell me about your experience using these rating scales? (Ease of use, confusion around meaning?)

o What were participants considering when they were rating difficulty of the task?

(Did it relate to just the physical task or concentrating on the task/ cognitive component)

o What were participants considering when they were rating your confidence to perform the task? (Whether they completed it, how it made them feel pleased, nervous etc.)

o What were you considering when they were rating difficulty of the task?

(Did it relate to just the physical task or concentrating on the task/ cognitive component)

o How did it feel progressing the task difficulty of the exercisses based on these ratings?

o Do you have any suggestions on how the rating scales could be improved?

**Technical difficulties with device**

- I understand that there were some technical difficulties with the device during your sessions. How did this impact your experience?

**Suitability**

- Who do you think the exciteBCI would be suitable for?
- Who is the exciteBCI not suitable for?
- Would a person using the exciteBCI need someone assisting or supervising to set-up and use the device, or could they manage it independently? How could this be improved?
- How long did the exciteBCI system take to set-up? Was this acceptable to you?

**Future use**

- Would you consider using the exciteBCI device in the future? If so, why? If not, why not?

*“I don’t have any further questions is there anything else you would to tell me about your experience?”*
